# Dibromido(2,3,9,10-tetra­methyl-1,4,8,11-tetra­azacyclo­tetra­deca-1,3,8,10-tetra­ene)cobalt(III) bromide

**DOI:** 10.1107/S160053680904166X

**Published:** 2009-10-17

**Authors:** Hoda El-Ghamry, Raafat Issa, Kamal El-Baradie, Shigeyuki Masaoka, Ken Sakai

**Affiliations:** a Department of Chemistry, Faculty of Science, Tanta University, Tanta, Egypt; b Department of Chemistry, Faculty of Science, Kyushu University, Hakozaki 6-10-1, Higashi-ku, Fukuoka 812-8581, Japan

## Abstract

In the title compound, [CoBr_2_(C_14_H_24_N_4_)]·Br, the Co^III^ ion is located on an inversion centre and possesses a distorted octa­hedral coordination geometry in which four nitro­gen donors of the ligand mol­ecule are in the equatorial plane and two Br^−^ ions occupy both the axial sites to give a *trans* isomer. The Br^- ^counter- anion is also located on an inversion centre.

## Related literature

For background to macrocyclic ligands and their metal complexes, see: Baird *et al.* (1993 ▶); Chandra & Verma (2008 ▶) and references therein; Chaudhary *et al.* (2002 ▶); Comba *et al.* (1986 ▶); Douglas (1978 ▶); Jones *et al.* (1979 ▶). For background to H_2_ evolution catalysis of macrocyclic metal complexes, see: Du *et al.* (2008 ▶); Fihri, Artero, Pereira & Fontecave (2008 ▶); Fihri, Artero, Raza­vet *et al.* (2008 ▶); Hu *et al.* (2007 ▶); Yamauchi *et al.* (2009 ▶). For the synthesis, see: Jackels *et al.* (1972 ▶).
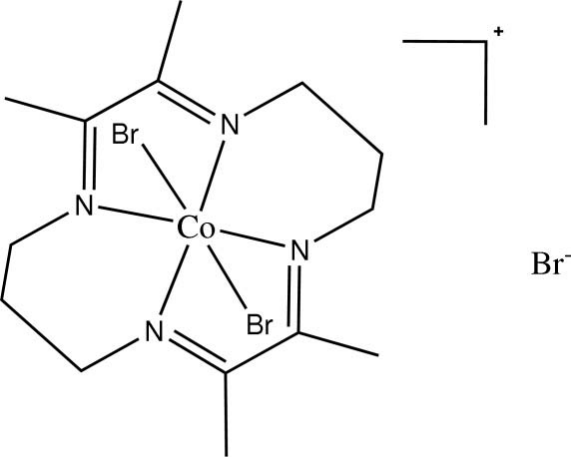


## Experimental

### 

#### Crystal data

[CoBr_2_(C_14_H_24_N_4_)]·Br
*M* *_r_* = 547.03
Triclinic, 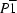

*a* = 7.3888 (10) Å
*b* = 7.5157 (10) Å
*c* = 8.1929 (11) Å
α = 84.647 (10)°
β = 84.760 (10)°
γ = 84.094 (10)°
*V* = 449.04 (10) Å^3^
*Z* = 1
Mo *K*α radiation
μ = 7.63 mm^−1^
*T* = 100 K
0.60 × 0.40 × 0.30 mm

#### Data collection

Bruker SMART APEXII CCD-detector diffractometer
Absorption correction: multi-scan (**SADABS**; Sheldrick, 1996 ▶) *T*_min_ = 0.045, *T*_max_ = 0.101
4629 measured reflections
1758 independent reflections
1739 reflections with *I* > 2σ(*I*)
*R*_int_ = 0.015

#### Refinement

*R*[*F* ^2^ > 2σ(*F* ^2^)] = 0.016
*wR*(*F* ^2^) = 0.040
*S* = 1.15
1758 reflections
105 parameters
H-atom parameters constrained
Δρ_max_ = 0.39 e Å^−3^
Δρ_min_ = −0.72 e Å^−3^

### 

Data collection: *APEX2* (Bruker, 2006 ▶); cell refinement: *SAINT* (Bruker, 2004 ▶); data reduction: *SAINT*; program(s) used to solve structure: *SHELXS97* (Sheldrick, 2008 ▶); program(s) used to refine structure: *SHELXL97* (Sheldrick, 2008 ▶); molecular graphics: *KENX* (Sakai, 2004 ▶); software used to prepare material for publication: *SHELXL97*, *TEXSAN* (Molecular Structure Corporation, 2001 ▶), *KENX* and *ORTEPII* (Johnson, 1976 ▶).

## Supplementary Material

Crystal structure: contains datablocks global, I. DOI: 10.1107/S160053680904166X/is2468sup1.cif

Structure factors: contains datablocks I. DOI: 10.1107/S160053680904166X/is2468Isup2.hkl

Additional supplementary materials:  crystallographic information; 3D view; checkCIF report

## Figures and Tables

**Fig. 1.**
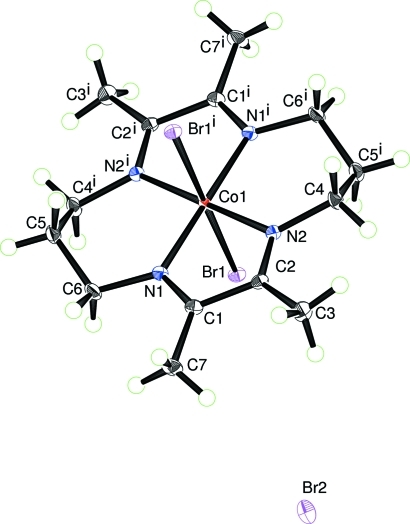
The molecular structure of (I) showing the atom-labeling scheme. Displacement ellipsoids are drawn at the 50% probability level.

**Fig. 2.**
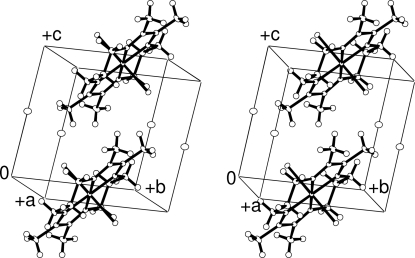
A stereoview for the crystal packing of (I).

## supplementary crystallographic information

### Comment

Most of the known synthetic macrocyclic ligands and their metal complexes have been prepared and characterized during the last few decades. Most commonly they are quadridentates containing nitrogen donor atoms, although compounds containing oxygen and sulfur donors are also known (Douglas, 1978). Metal template synthesis of multidentate and macromonocyclic ligands have been established over the last three decades as offering high yield and selective routes to new ligands and their complexes (Comba *et al.*, 1986). Transition metal macrocyclic complexes have received much attention as active part of metalloenzymes (Chaudhary *et al.*, 2002) as biomimic model compounds (Jones *et al.*, 1979) due to their resemblance with natural proteins like hemerythrin and enzymes. They also played an important role as catalysts in oxidation and epoxidation processes (Chandra *et al.*, 2008). There are some recent reports about some macrocyclic Co^II^ and Co^III^ complexes which showed high activity towards H_2_ evolution electrochemically (Hu *et al.*, 2007) or photochemically (Fihri, Artero, Pereira & Fontecave, 2008; Fihri, Artero, Razavet *et al.*, 2008; Du *et al.*, 2008). The title compound has been observed to evolve H_2_ electrocatalytically in acetonitrile (Hu *et al.*, 2007). Unfortunately, it is found that this compound does not show any catalytic activity towards H_2_ evolution in a well known photosystem consisting of tris (2,2^'^-bipyridine)ruthenium(II) as a photosensitizer, methylviologen (*N*,*N*^'^-dimethyl-4,4^'^-bipyridinium) as an electron mediator, and ethylenediaminetetraacetic acid disodium salt as a sacrificial electron donor. Because of our on-going studies on the H_2_-evolving activity of Pt^II^ based molecular catalysts (Yamauchi *et al.*, 2009), attempts have been made to obtain the Pt^II^ complex of the present macrocyclic ligand. However the metal exchange from Co^III^ to Pt^II^ has been unsuccessful so far, presunably due to the extremely high stability of the Co^III^ complex, during the course of these studies we have succeeded in the *x*-ray crystal structure determination of the present compound.

The Co^III^ ion and the Br^-^ ion involved as a counter anion are respectively located at crystallographic inversion centers. Because of these requirements four nitrogen donors, two of them are independent, comprise a crystalloaphically planar geometry and the Co^III^ ion is also located exactly on the same plane. The vector defined by the Co—Br bond is slightly declined from the vector which is peependicular to the basal plane consisting of the four nitrogen donor atoms which can be recognized from the N—Co—Br angles; [N2—Co1—Br1=91.78 (4)° and N1—Co1—Br1=89.14 (4)°]. It is also observed that the Co—N, N═C and N—C bond distances of 1.9208 (13), 1.288 (2) and 1.472 (2) Å, respectively, are in accordance with the reported values for similar Co^III^ imine type macrocyclic complexes [2,9-dimethyl-3,10- diphenyl-1,4,8,11-tetraazacyclotetradeca-1,3,8,10-tetraene)cobalt(III); Co—N, N═C and N—C distances are 1.923 (13), 1.278 (3) and 1.478 (3) Å, respectively] (Baird *et al.*, 1993). The N2, C4, C5^i^, C6^i^ and N1^i^ atoms form an envelope geometry in which the triangle defined by atoms C4, C5^i^ and C6^i^ is canted by 60.77 (11)° with respect to the least square plane defined by N1^i^, C6^i^, C4 and N2 atoms [symmetry code: (i) -*x* + 1, -*y* + 1, -*z* + 2]. No remarkable intercationic or cation-anion interactions are found in the crystal.

### Experimental

The title compound was synthesized according to the method reported by Jackels *et al.* (1972). Elemental analysis calculated for C_14_H_24_N_4_Br_3_Co: C 30.74, H 4.42, N 10.42%. Found: C 30.40, H 4.50, N 10.04%. ESI-TOF MS (positive ion, methanol): m/*z* 466.9 [*M*^+^]. IR (ν, cm^-1^): 3204(w), 2980(*m*), 2933(*s*), 2889(*s*), 2766(w), 2005(*m*), 1615(w), 1597(*m*), 1476(*m*), 1461(*s*), 1426(*m*), 1408(*m*), 1372(w), 1333(w), 1288(*m*), 1214(*s*), 1187(*m*), 1026(*m*), 938(*s*), 868(*m*), 831(w), 806(w), 777(*s*), 560(w), 444(*s*). Recrystallization of the crude product by a method reported in the same paper resulted in the formation of dark green crystals suitable for X-ray diffraction analysis.

### Refinement

All H atoms were placed in idealized positions (methyl C—H = 0.96 Å, methylene C—H = 0.97 Å), and included in the refinement in a riding-model approximation, with *U*_iso_(H) =1.5*U*_eq_(methyl C) and *U*_iso_(H) =1.2*U*_eq_(methylene C). In the final difference Fourier map, the highest peak was located 0.97 Å from atom Br1. The deepest hole was located 1.92 Å from atom Br1.

### Crystal data

**Table:**

[CoBr_2_(C_14_H_24_N_4_)]·Br	*Z* = 1
*M**_r_* = 547.03	*F*(000) = 268
Triclinic, *P*1	? # Insert any comments here.
Hall symbol: -P 1	*D*_x_ = 2.023 Mg m^−^^3^
*a* = 7.3888 (10) Å	Mo *K*α radiation, λ = 0.71073 Å
*b* = 7.5157 (10) Å	Cell parameters from 4684 reflections
*c* = 8.1929 (11) Å	θ = 2.5–28.3°
α = 84.647 (10)°	µ = 7.63 mm^−^^1^
β = 84.76 (1)°	*T* = 100 K
γ = 84.094 (10)°	Brocks, dark green
*V* = 449.04 (10) Å^3^	0.60 × 0.40 × 0.30 mm

### Data collection

**Table:**

Bruker SMART APEX CCD-detector diffractometer	1758 independent reflections
Radiation source: sealed tube	1739 reflections with *I* > 2σ(*I*)
graphite	*R*_int_ = 0.015
φ and ω scans	θ_max_ = 26.0°, θ_min_ = 2.5°
Absorption correction: multi-scan (*SADABS*; Sheldrick, 1996)	*h* = −9→9
*T*_min_ = 0.045, *T*_max_ = 0.101	*k* = −9→9
4629 measured reflections	*l* = −10→10

### Refinement

**Table:**

Refinement on *F*^2^	Secondary atom site location: difference Fourier map
Least-squares matrix: full	Hydrogen site location: inferred from neighbouring sites
*R*[*F*^2^ > 2σ(*F*^2^)] = 0.016	H-atom parameters constrained
*wR*(*F*^2^) = 0.040	*w* = 1/[σ^2^(*F*_o_^2^) + (0.0205*P*)^2^ + 0.2736*P*] where *P* = (*F*_o_^2^ + 2*F*_c_^2^)/3
*S* = 1.15	(Δ/σ)_max_ = 0.001
1758 reflections	Δρ_max_ = 0.39 e Å^−^^3^
105 parameters	Δρ_min_ = −0.72 e Å^−^^3^
0 restraints	Extinction correction: *SHELXL*
Primary atom site location: structure-invariant direct methods	Extinction coefficient: 0.036 (4)

### Special details

**Table:**

Experimental. The first 50 frames were rescanned at the end of data collection to evaluate any possible decay phenomenon. Since it was judged to be negligible, no decay correction was applied to the data.
Geometry. All e.s.d.'s (except the e.s.d. in the dihedral angle between two l.s. planes) are estimated using the full covariance matrix. The cell e.s.d.'s are taken into account individually in the estimation of e.s.d.'s in distances, angles and torsion angles; correlations between e.s.d.'s in cell parameters are only used when they are defined by crystal symmetry. An approximate (isotropic) treatment of cell e.s.d.'s is used for estimating e.s.d.'s involving l.s. planes.Least-squares planes (*x*,*y*,*z* in crystal coordinates) and deviations from them (* indicates atom used to define plane)4.5770 (0.0058) *x* + 4.5950 (0.0059) *y* - 3.7053 (0.0042) *z* = 0.8322 (0.0063)* 0.0209 (0.0009) C4 * -0.0208 (0.0009) C6_$1 * -0.0182 (0.0008) N2 * 0.0181 (0.0008) N1_$1Rms deviation of fitted atoms = 0.0195- 2.0878 (0.0161) *x* + 6.6317 (0.0050) *y* - 1.8850 (0.0100) *z* = 2.7313 (0.0098)Angle to previous plane (with approximate e.s.d.) = 60.77 (0.11)* 0.0000 (0.0000) C4 * 0.0000 (0.0000) C5_$1 * 0.0000 (0.0000) C6_$1Rms deviation of fitted atoms = 0.0000
Refinement. Refinement of *F*^2^ against ALL reflections. The weighted *R*-factor *wR* and goodness of fit *S* are based on *F*^2^, conventional *R*-factors *R* are based on *F*, with *F* set to zero for negative *F*^2^. The threshold expression of *F*^2^ > σ(*F*^2^) is used only for calculating *R*-factors(gt) *etc*. and is not relevant to the choice of reflections for refinement. *R*-factors based on *F*^2^ are statistically about twice as large as those based on *F*, and *R*- factors based on ALL data will be even larger.

### Fractional atomic coordinates and isotropic or equivalent isotropic displacement parameters (Å^2^)

**Table:**

	*x*	*y*	*z*	*U*_iso_*/*U*_eq_	
Br1	0.32441 (2)	0.29517 (2)	1.169271 (18)	0.01131 (7)	
Br2	0.0000	0.0000	0.5000	0.01880 (8)	
Co1	0.5000	0.5000	1.0000	0.00682 (8)	
N1	0.57154 (18)	0.31635 (18)	0.85246 (16)	0.0103 (3)	
N2	0.30942 (18)	0.55749 (18)	0.85385 (16)	0.0097 (3)	
C1	0.4642 (2)	0.3094 (2)	0.7383 (2)	0.0117 (3)	
C2	0.3142 (2)	0.4563 (2)	0.7350 (2)	0.0109 (3)	
C3	0.1898 (2)	0.4778 (3)	0.5990 (2)	0.0169 (4)	
H3A	0.1135	0.3808	0.6109	0.025*	
H3B	0.2612	0.4768	0.4951	0.025*	
H3C	0.1150	0.5898	0.6038	0.025*	
C4	0.1733 (2)	0.7132 (2)	0.8684 (2)	0.0143 (3)	
H4A	0.0653	0.6924	0.8167	0.017*	
H4B	0.2226	0.8187	0.8109	0.017*	
C5	0.8791 (2)	0.2526 (2)	0.9535 (2)	0.0145 (3)	
H5A	0.9114	0.3638	0.8933	0.017*	
H5B	0.9864	0.1668	0.9480	0.017*	
C6	0.7296 (2)	0.1820 (2)	0.8706 (2)	0.0148 (3)	
H6A	0.7777	0.1473	0.7628	0.018*	
H6B	0.6902	0.0758	0.9349	0.018*	
C7	0.4802 (3)	0.1698 (2)	0.6181 (2)	0.0174 (4)	
H7A	0.5370	0.2163	0.5150	0.026*	
H7B	0.3608	0.1382	0.6021	0.026*	
H7C	0.5531	0.0652	0.6598	0.026*	

### Atomic displacement parameters (Å^2^)

**Table:**

	*U*^11^	*U*^22^	*U*^33^	*U*^12^	*U*^13^	*U*^23^
Br1	0.01353 (10)	0.01049 (10)	0.01015 (10)	−0.00325 (6)	−0.00175 (6)	0.00102 (6)
Br2	0.01990 (13)	0.01933 (14)	0.01523 (13)	0.00443 (10)	0.00017 (9)	0.00027 (10)
Co1	0.00848 (15)	0.00660 (14)	0.00573 (15)	−0.00006 (11)	−0.00254 (11)	−0.00114 (11)
N1	0.0123 (7)	0.0092 (6)	0.0097 (6)	−0.0010 (5)	−0.0021 (5)	−0.0005 (5)
N2	0.0107 (6)	0.0098 (6)	0.0085 (6)	−0.0012 (5)	−0.0019 (5)	0.0003 (5)
C1	0.0148 (8)	0.0110 (7)	0.0096 (7)	−0.0023 (6)	−0.0010 (6)	−0.0010 (6)
C2	0.0122 (8)	0.0120 (7)	0.0091 (7)	−0.0035 (6)	−0.0022 (6)	0.0002 (6)
C3	0.0185 (9)	0.0210 (9)	0.0125 (8)	0.0014 (7)	−0.0081 (7)	−0.0044 (7)
C4	0.0145 (8)	0.0140 (8)	0.0143 (8)	0.0047 (6)	−0.0057 (6)	−0.0020 (6)
C5	0.0133 (8)	0.0136 (8)	0.0162 (8)	0.0036 (6)	−0.0031 (6)	−0.0020 (7)
C6	0.0168 (8)	0.0120 (8)	0.0162 (8)	0.0038 (6)	−0.0046 (7)	−0.0063 (6)
C7	0.0232 (9)	0.0159 (8)	0.0147 (8)	0.0005 (7)	−0.0058 (7)	−0.0077 (7)

### Geometric parameters (Å, °)

**Table:**

Br1—Co1	2.3792 (2)	C3—H3C	0.9600
Co1—N2	1.9208 (13)	C4—H4A	0.9700
Co1—N1	1.9210 (13)	C4—H4B	0.9700
N1—C1	1.288 (2)	C5—C6	1.513 (2)
N1—C6	1.472 (2)	C5—H5A	0.9700
N2—C2	1.286 (2)	C5—H5B	0.9700
N2—C4	1.469 (2)	C6—H6A	0.9700
C1—C2	1.482 (2)	C6—H6B	0.9700
C1—C7	1.494 (2)	C7—H7A	0.9600
C2—C3	1.495 (2)	C7—H7B	0.9600
C3—H3A	0.9600	C7—H7C	0.9600
C3—H3B	0.9600		
			
N2^i^—Co1—N2	180.000 (1)	C2—C3—H3C	109.5
N2—Co1—N1^i^	98.31 (6)	H3A—C3—H3C	109.5
N2^i^—Co1—N1	98.31 (6)	H3B—C3—H3C	109.5
N2—Co1—N1	81.69 (6)	N2—C4—H4A	109.3
N1^i^—Co1—N1	180.0	C5^i^—C4—H4A	109.3
N2—Co1—Br1^i^	88.22 (4)	N2—C4—H4B	109.3
N1—Co1—Br1^i^	90.86 (4)	C5^i^—C4—H4B	109.3
N2^i^—Co1—Br1	88.22 (4)	H4A—C4—H4B	108.0
N2—Co1—Br1	91.78 (4)	C6—C5—H5A	108.8
N1^i^—Co1—Br1	90.86 (4)	C4^i^—C5—H5A	108.8
N1—Co1—Br1	89.14 (4)	C6—C5—H5B	108.8
C1—N1—C6	120.39 (14)	C4^i^—C5—H5B	108.8
C1—N1—Co1	115.40 (11)	H5A—C5—H5B	107.7
C6—N1—Co1	124.08 (10)	N1—C6—C5	112.00 (13)
C2—N2—C4	121.39 (14)	N1—C6—H6A	109.2
C2—N2—Co1	115.56 (11)	C5—C6—H6A	109.2
C4—N2—Co1	122.93 (11)	N1—C6—H6B	109.2
N1—C1—C2	113.55 (14)	C5—C6—H6B	109.2
N1—C1—C7	125.76 (15)	H6A—C6—H6B	107.9
C2—C1—C7	120.68 (14)	C1—C7—H7A	109.5
N2—C2—C1	113.57 (14)	C1—C7—H7B	109.5
N2—C2—C3	126.51 (15)	H7A—C7—H7B	109.5
C1—C2—C3	119.89 (14)	C1—C7—H7C	109.5
C2—C3—H3A	109.5	H7A—C7—H7C	109.5
C2—C3—H3B	109.5	H7B—C7—H7C	109.5
H3A—C3—H3B	109.5		
			
C6—N1—C1—C2	−178.89 (14)	C7—C1—C2—N2	174.67 (15)
Co1—N1—C1—C2	5.11 (18)	N1—C1—C2—C3	173.64 (15)
C6—N1—C1—C7	1.7 (3)	C7—C1—C2—C3	−6.9 (2)
Co1—N1—C1—C7	−174.28 (13)	C2—N2—C4—C5^i^	148.20 (15)
C4—N2—C2—C1	178.26 (14)	Co1—N2—C4—C5^i^	−35.93 (19)
Co1—N2—C2—C1	2.11 (18)	C1—N1—C6—C5	154.97 (15)
C4—N2—C2—C3	0.0 (3)	Co1—N1—C6—C5	−29.39 (19)
Co1—N2—C2—C3	−176.16 (14)	C4^i^—C5—C6—N1	66.89 (18)
N1—C1—C2—N2	−4.8 (2)		

Symmetry codes: (i) −*x*+1, −*y*+1, −*z*+2.
